# Effect of age and disease on bone mass in Japanese patients with schizophrenia

**DOI:** 10.1186/1744-859X-11-5

**Published:** 2012-02-20

**Authors:** Norio Sugawara, Norio Yasui-Furukori, Takashi Umeda, Shoko Tsuchimine, Akira Fujii, Yasushi Sato, Manabu Saito, Hanako Furukori, Kazuma Danjo, Masashi Matsuzaka, Ippei Takahashi, Sunao Kaneko

**Affiliations:** 1 Department of Neuropsychiatry, Hirosaki University School of Medicine, Hirosaki, Japan; 2 Department of Social Medicine, Hirosaki University School of Medicine, Hirosaki, Japan; 3 Department of Psychiatry, Hirosaki-Aiseikai Hospital, Hirosaki, Japan; 4 Department of Psychiatry, Kuroishi-Akebono Hospital, Kuroishi, Japan

**Keywords:** bone mass, Japanese, schizophrenia, ultrasound

## Abstract

**Background:**

There have been a limited number of studies comparing bone mass between patients with schizophrenia and the general population. The aim of this study was to compare the bone mass of schizophrenia patients with that of healthy subjects in Japan.

**Methods:**

We recruited patients (n = 362), aged 48.8 ± 15.4 (mean ± SD) years who were diagnosed with schizophrenia or schizoaffective disorder based on the *Diagnostic and Statistical Manual of Mental Disorders*, fourth edition (DSM-IV). Bone mass was measured using quantitative ultrasound densitometry of the calcaneus. The osteosono-assessment index (OSI) was calculated as a function of the speed of sound and the transmission index. For comparative analysis, OSI data from 832 adults who participated in the Iwaki Health Promotion Project 2009 was used as representative of the general community.

**Results:**

Mean OSI values among male schizophrenic patients were lower than those in the general population in the case of individuals aged 40 and older. In females, mean OSI values among schizophrenic patients were lower than those in the general community in those aged 60 and older. In an analysis using the general linear model, a significant interaction was observed between subject groups and age in males.

**Conclusions:**

Older schizophrenic patients exhibit lower bone mass than that observed in the general population. Our data also demonstrate gender and group differences among schizophrenic patients and controls with regard to changes in bone mass associated with aging. These results indicate that intervention programs designed to delay or prevent decreased bone mass in schizophrenic patients might be tailored according to gender.

## Introduction

It has previously been shown that patients taking antipsychotic medications are at greater risk for bone fractures [1]. The onset of schizophrenia typically occurs during adolescence and young adulthood [2], therefore, the administration of antipsychotic medications generally begins during the same period, during which bone maturation results in peak bone mass.

In previous studies that have considered decreased bone mass in patients with schizophrenia, factors associated with accelerated bone absorption have included polydipsia [3], use of neuroleptics [4] and the resulting hyperprolactinemia [5,6], heavy smoking [7], poor diet, drug and alcohol abuse [8], and lack of exercise [9].

However, to date there have been a limited number of studies [10-12] comparing bone mass between patients with schizophrenia and the general population. It is therefore necessary to accurately examine the nature of bone mass deficiencies in schizophrenic patients in a cross-sectional manner with an age-matched healthy control group for comparison.

In recent years, a quantitative ultrasound (QUS) densitometry technique has been developed for measuring bone mass. The results obtained using QUS are reported to correlate well with those using dual energy x-ray absorptiometry (DXA) [13]. Furthermore, QUS can provide data on the risk for osteoporosis related fractures [14,15]. QUS is a relatively quick procedure that is inexpensive and does not expose patients to radiation, making it suitable for research projects examining large numbers of subjects [16].

The aim of this study was to compare bone mass between patients with schizophrenia and healthy individuals using QUS. To the best of our knowledge, this is the largest study of this nature to be carried out in Japan.

## Methods

### Participants

The subject pool consisted of 362 patients (178 males and 184 females) who were diagnosed with either schizophrenia or schizoaffective disorder based on *Diagnostic and Statistical Manual of Mental Disorders*, fourth edition (DSM-IV) criteria at 3 psychiatric hospitals in Japan. The diagnoses of the patients were determined based on medical records. As a reference group, 832 healthy volunteers (327 males and 505 females) who participated in the Iwaki Health Promotion Project in 2009 were also included. They were residents of Iwaki district, Hirosaki city, in northern Japan. Iwaki district is a stable community with a population 11, 961. Their demographic data (age and sex) and medical history were obtained from self-report questionnaires and interviews. Data obtained from the meteorological agency website showed that environmental conditions (duration of sunshine, length of day, temperature and humidity) were similar in the three hospitals and control survey [17]. The data collection procedures for this study were approved (no. 2008-122) by the Ethics Committee of the Hirosaki University School of Medicine and all subjects provided written informed consent before participating.

### Procedure

Subject demographic data (age and sex) was obtained from their medical records. Bone mass was evaluated via QUS densitometry using the AOS-100 device (Aloka, Co. Ltd., Tokyo, Japan) at the calcaneus. The AOS-100 evaluates the speed of sound (SOS in m/s) and the transmission index (TI). SOS and TI as measured using the AOS-100, are highly correlated with SOS (r = 0.89) and broadband ultrasound attenuation (BUA) (r = 0.88) measures obtained using a conventional QUS device [18]. The osteosono-assessment index (OSI) was calculated as a function of SOS and TI, using the TI value multiplied by the square of the SOS value [19]. We assessed the precision of quantitative ultrasound densitometry, and intratest precision was calculated from 3 repeated scans with repositioning in 17 volunteers; the short-term coefficient of variation was 2.6% for the OSI measure [20]. Low bone mass was defined as a level equal to or less than -1.0 standard deviation below the mean OSI value of the reference group aged 20 to 39 years old. Severe low bone mass was defined as a level equal to or less than -2.5 standard deviations below the mean OSI value of the reference group.

### Statistical analysis

Subjects were classified into two groups, with recruited schizophrenic patients composing the schizophrenic patient group and community residents composing the general population group. To compare the main demographic and clinical characteristics between schizophrenic patients and the community group, an unpaired Student's t test was performed to analyze continuous variables, and a χ^2 ^test or Fisher's exact test was used for categorical variables. Data are presented as mean ± SD. Subjects were classified into six age groups, with those aged 20 to 69 grouped according to decade and one group for those 70 and older. In addition, subjects were classified into two larger groups (the younger group with those aged 20 to 49, and the older group for those aged 50 and older) to analyze the effect of aging. The relationship between OSI values and age was tested using simple linear regression analysis. To determine the factors associated with the measured OSI value, a general linear model analysis that included age and subject groups was employed. Interactions between age and subject group were also tested in the same model. A value of *P *< 0.05 was considered significant. The data were analyzed using PASW Statistics software for Windows, V. 18.0.0 (SPSS Inc., Chicago, IL, USA).

## Results

### Demographic characteristics

The demographic data and OSI values separated by subject group, age groups and gender are presented in Table 1. The mean age was 48.0 ± 14.9 years in the male patients and 49.7 ± 15.9 years in female patients. In the general population group, the mean age was 56.0 ± 13.8 years in males and 58.1 ± 13.0 years in females. Mean OSI values were lower in male schizophrenic patients compared with males in the community group in individuals 40 and older. In females, mean OSI values were also lower for schizophrenic patients compared with the community group in those 60 and older. Intragroup analysis as comparisons between younger and older group has shown significantly lower OSI values of the older group in both male schizophrenic patients (*P *< 0.001) and community group (*P *< 0.01). Among females, the older group also showed significantly lower OSI values than the younger group in both schizophrenic patients (*P *< 0.001) and community group (*P *< 0.001).

**Table 1:** Basic characteristics of schizophrenic patients compared to the general community in Japanese subjects

Age subgroup, years	Subjects		OSI	
	
	Schizophrenia, n	Community, n	Schizophrenia, mean (SD)	Community, mean (SD)
Male:				
20 to 29	20	15	3.13 ± 0.35	2.97 ± 0.52
30 to 39	43	28	2.89 ± 0.49	3.06 ± 0.46
40 to 49	32	57	2.76 ± 0.55*	2.96 ± 0.38
50 to 59	40	90	2.68 ± 0.43*	2.86 ± 0.32
60 to 69	30	76	2.57 ± 0.56*	2.87 ± 0.40
70 or older	13	61	2.10 ± 0.37*	2.84 ± 0.51
Total	178	327		
Female:				
20 to 29	24	15	2.80 ± 0.38	2.88 ± 0.34
30 to 39	32	40	2.98 ± 0.67	2.85 ± 0.37
40 to 49	34	62	2.72 ± 0.36	2.74 ± 0.32
50 to 59	39	136	2.48 ± 0.28	2.55 ± 0.36
60 to 69	28	150	2.20 ± 0.30*	2.37 ± 0.24
70 or older	27	102	2.08 ± 0.22*	2.27 ± 0.23
Total	184	505		

Student's unpaired t test was used to evaluate the differences of osteosono-assessment index (OSI) values between schizophrenic patients and general community. *P < 0.05.

**Figure 1:**
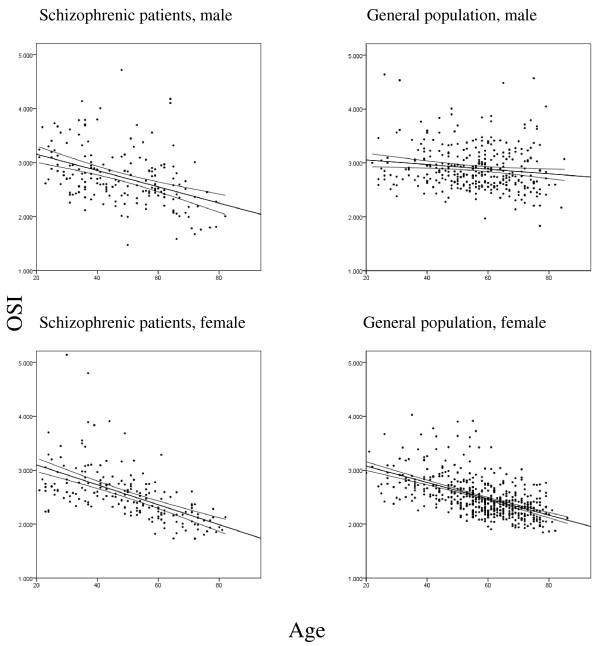
**Scatterplots depicting the relationship between osteosono-assessment index (OSI) and age across subject populations and genders**. Lines indicate the slope of best fit with a 95% confidence interval by simple linear regression analysis.

Trends in OSI values are depicted in Figure 1. A significant relationship between mean OSI value and age was observed in both genders (male, *P *< 0.001; female, *P *< 0.001) for schizophrenic patients. The trend was also significant among males (*P *< 0.01) and females (*P *< 0.001) in the general population.

### Prevalence of low bone mass

Table 2 shows the prevalence of low bone mass (t score < -1 computed from OSI value) and severe low bone mass (t score < -2.5 computed from OSI value) in schizophrenic patient group compared to the community group. In males, the prevalence of low bone mass among schizophrenic patients is higher than that in the community group among age groups beginning at 30 years old. Furthermore, the prevalence of severe low bone mass among schizophrenic patients is higher than in community group in the 70 and older age group. In females, the prevalence of severe low bone mass among schizophrenic patients is higher than that in the community group for age groups 60 and older.

**Table 2:** Comparison of the prevalence of low bone mass in schizophrenic patients versus the general community in Japanese subjects

Age subgroup, years	Prevalence of low bone mass (t < 1.0)		Prevalence of severe low bone mass (t < 2.5)	
	
	Schizophrenia, %	Community, %	Schizophrenia, %	Community, %
Male:				
20 to 29	0(0/20)	6.7 (1/15)	0(0/20)	0 (0/15)
30 to 39	27.9(12/43)*	7.1 (2/28)	0(0/43)	0 (0/28)
40 to 49	34.4(11/32)*	8.8 (5/57)	0(0/32)	0 (0/57)
50 to 59	42.5(17/40)*	12.2 (11/90)	2.5(1/40)	0 (0/90)
60 to 69	60(18/30)*	17.1 (13/76)	3.3(1/30)	0 (0/76)
70 or older	92.3(12/13)*	31.1 (19/61)	30.8(4/13)*	0 (0/61)
Female:				
20 to 29	16.7(4/24)	13.3 (2/15)	0(0/24)	0 (0/15)
30 to 39	21.9(7/32)	15.0 (6/40)	0(0/32)	0 (0/40)
40 to 49	23.5(8/34)	24.2 (15/62)	0(0/34)	0 (0/62)
50 to 59	51.3(20/39)	54.4 (74/136)	2.6(1/39)	0 (0/136)
60 to 69	85.7(24/28)	78.0 (117/150)	7.1(2/28)*	0 (0/150)
70 or older	96.3(26/27)	85.3 (87/102)	14.8(4/27)*	1.0 (1/102)

The obtained data were analyzed using a χ^2 ^test or Fisher's exact test between schizophrenic patients and general community. *P < 0.05

### Comparison of OSI with age and disease status in each gender using general linear model

Table 3 shows the general linear model results for the association between subject group, age, and OSI. In males, OSI values in schizophrenic patients are significantly lower than those in the general population (*P *< 0.05). A significant effect of age on OSI value is also observed. Furthermore, a significant interaction can be observed between subject group and age in males. In females, a significant effect of age on OSI value is observed.

**Table 3:** Comparison of osteosono-assessment index (OSI) by age and disease in each gender with general linear model

	Male			Female		
		
	Coefficient	SE	*P *value	Coefficient	SE	*P *value
Group (being schizophrenic)	-0.318	0.149	*P *< 0.05	-0.091	0.104	*P *= 0.383
Age	-0.015	0.002	*P *< 0.001	-0.019	0.001	*P *< 0.001
Group × age	0.011	0.003	*P *< 0.001	0.003	0.002	*P *= 0.079
Constant	3.459	0.110	*P *< 0.001	3.471	0.079	*P *< 0.001

To determine the factors associated with the measured OSI value, a general linear model that included subject groups (being schizophrenic), age, and interaction (subject groups × age) was employed.

## Discussion

This study was designed to evaluate the effects of age and disease on bone mass in patients diagnosed with schizophrenia. In both males and females, schizophrenic patients had lower bone mass than that of the general population in age groups of 60 years and older. A descending trend between mean OSI value and age was observed in both genders in the schizophrenic patients and in the community group. However, a significant interaction between subject group and age in general linear model showed that descending trend of OSI value with aging was accelerated in male schizophrenic patients.

Several previous studies have compared bone mass between schizophrenic patients and the general population. In a large Taiwanese study, Renn *et al*. [11] reported that 965 schizophrenic patients had lower bone mass values than those observed in the general population under the age of 50 in both genders. However, bone mass was higher in schizophrenic patients over the age of 60 than in the general population in both genders, indication that an aging effect on bone mass was not observed in schizophrenic patients. However, Kishimoto *et al*. [10] reported that bone mass among 74 male schizophrenic patients was lower than in the general population in the age group of 40 to 49 and 55 to 79. Furthermore, Jung *et al*. [12] also reported that 229 schizophrenic patients over age 50 had a higher prevalence of osteoporosis than healthy controls. In addition to the comparison studies with the general population, Liu-Seifert *et al*. [21] reported that low bone mineral density (BMD) was significantly associated with age in both genders among schizophrenic patients.

One possible explanation for the discrepancies observed regarding aging effects on bone mass among older schizophrenic patients is that those with the greatest bone fracture risk may expire at a relatively young age, leaving a cohort of survivors in the patient group over 60 years of age in the Taiwanese study. Another possible explanation is that the location from which the patients and controls were recruited differed in the Taiwanese study. In the current study, we recruited both patients and controls from the Aomori prefecture to rule out any geography-related effects.

In this study, the trend relating OSI value and aging among schizophrenic patients does not differ from that in the general population in females. One possible explanation is that the effects of menopause on bone loss might exceed those of antipsychotic medication or the disease itself. Estrogen deficiency at menopause leads to increased skeletal remodeling and loss of bone mass [22]. A decrease in estrogen levels can influence the activity of interleukins, which are known to influence dynamic bone homeostasis [23]. The protective effect of estrogen to bone mass might explain the earlier decline of bone mass among male schizophrenic patients than female ones. Another possible explanation is that secretion of estrogen or leptin from stored bodily fat might protect bone loss in female patients with schizophrenia, as these patients have more body fat [24,25] than the general population. Previous studies [26,27] have additionally shown a relationship between fat mass and bone mineral density among females.

The mechanism for decreased bone mass among patients with schizophrenia has not been entirely elucidated. However, most antipsychotics block dopamine-D2 receptors, attenuating the inhibitory effect of dopamine on prolactin release from the pituitary gland and resulting in hyperprolactinemia [28]. Previous studies have shown that patients who are treated with antipsychotics have elevated prolactin levels [6,29], which is correlated with decreased bone mass in schizophrenic patients. Even when a direct effect of prolactin on BMD was not observed, the duration of antipsychotic treatment had a tendency to be associated with decreased BMD [30], and patients with hyperprolactinemia showed higher rates of bone metabolism, including both bone formation and resorption [5].

The present study has some limitations. First, measurement of bone mass was based on QUS densitometry instead of DXA scans. There are many researchers who question the precision of peripheral bone mineral density measurements. Previous studies have reported that QUS parameters cannot be used to predict decreased BMD and that the sensitivities and specificities of QUS parameters are not sufficient to allow it to be used as an alternative to DXA [31-33], which is currently the gold standard for measuring BMD [34,35]. However, most psychiatric clinics do not have access to DXA equipment and DXA scanning is time consuming, costly and exposes patients to radiation, rendering it less than ideal for a large population survey. In contrast, QUS is rapid and portable, making it a suitable method for large studies. For these reasons, we chose to use this method for our study. Second, because all participants of patients and control consisted of volunteers, they may have more interests in their health or be healthier than the outpatient population or general population. Third, not all parameters which could affect bone mass were included in this study such as body mass index, dietary habits, smoking, alcohol intake, physical exercise, duration of illness and treatment, schizophrenic symptoms and medications. Antipsychotic medications in particular may be an important factor, as the use of prolactin-sparing or prolactin-raising antipsychotics might confound the results. Future research should endeavor to consider the effects of drug treatment and lifestyle factors in conjunction with decreased bone mass in schizophrenia.

## Conclusions

This study has shown that the patients with schizophrenia have lower bone mass than the general population in older age groups. However, the descending trend between bone mass and aging among male schizophrenic patients is different from that observed in the male community. This suggests that intervention programs to either prevent or delay the onset of osteoporosis among schizophrenic patients might be tailored separately for each gender.

## Competing interests

The authors declare that they have no competing interests.

## Authors' contributions

NS conceived the study, designed the study, conducted the statistical analysis, interpreted the data and wrote the initial draft of the manuscript. SK and NYF had full access to all of the data in the study and take responsibility for the integrity of the data and the accuracy of the data analysis. ST, YS and MS contributed to study design and assisted in drafting the manuscript. AF and HF completed initial survey construction, recruitment of participants. TU, IT, MM and KD participated in the data collection, and the interpretation of the results. All authors approved the final manuscript.
